# Chemokines, selectins and intracellular calcium flux: temporal and spatial cues for leukocyte arrest

**DOI:** 10.3389/fimmu.2012.00188

**Published:** 2012-07-10

**Authors:** Neha Dixit, Scott I. Simon

**Affiliations:** Department of Biomedical Engineering, Graduate Group in Immunology, University of California, DavisCA, USA

**Keywords:** calcium, chemokine, cytoskeletal proteins, inflammation, integrin affinity, LFA-1, neutrophils, Orai1

## Abstract

Leukocyte trafficking to acute sites of injury or infection requires spatial and temporal cues that fine tune precise sites of firm adhesion and guide migration to endothelial junctions where they undergo diapedesis to sites of insult. Many detailed studies on the location and gradient of chemokines such as IL-8 and other CXCR ligands reveal that their recognition shortly after selectin-mediated capture and rolling exerts acute effects on integrin activation and subsequent binding to their ligands on the endothelium, which directs firm adhesion, adhesion strengthening, and downstream migration. In this process, G-protein coupled receptor (GPCR) signaling has been found to play an integral role in activating and mobilizing intracellular stores of calcium, GTPases such as Rap-1 and Rho and cytokeletal proteins such as Talin and F-actin to facilitate cell polarity and directional pseudopod formation. A critical question remaining is how intracellular Ca^2+^ flux from CRAC channels such as Orai1 synergizes with cytosolic stores to mediate a rapid flux which is critical to the onset of PMN arrest and polarization. Our review will highlight a specific role for calcium as a signaling messenger in activating focal clusters of integrins bound to the cytoskeleton which allows the cell to attain a migratory phenotype. The precise interplay between chemokines, selectins, and integrins binding under the ubiquitous presence of shear stress from blood flow provides an essential cooperative signaling mechanism for effective leukocyte recruitment.

## Triggering leukocyte adhesion at vascular sites of inflammation

Leukocyte recruitment to sites of inflammatory insult has been described as a multi-step process governed by chemokines, selectins, and integrins that engage in a step-wise manner to initiate intracellular signals and adhesive bond formation (Campbell et al., 1998; Ley, 2002; Simon and Green, 2005). β_2_-integrins are key adhesion receptors in this process as they perform both adhesion and signaling functions. In the circulation, β_2_-integrins are expressed on the membrane at low numbers and in a low affinity state that rapidly shift to high affinity and increase in number, and surface density as they make contact with endothelium at sites of inflammation. Affinity is regulated via allosteric changes in integrin structure that in turn modulate their adhesion potential. Following selectin dependent capture and rolling, an upshift occurs from a low affinity bent conformation to an extended conformation associated with intermediate affinity that can bind to endothelial ligands and effect deceleration of rolling leukocytes. Chemokines play a key role in signaling a shift in integrin conformation from intermediate to high affinity that is associated with adhesive stabilization, such that the leukocyte becomes resistant to tensile and shear repulsive forces of blood flow. In fact, it is control of the number and density of high affinity integrins and endothelial presentation of their cognate ligands that determines when and where leukocytes are recruited to emigrate during inflammation (Constantin et al., 2000; Beals et al., 2001; Kim et al., 2004; Sarantos et al., 2005; Bachmann et al., 2006; Green et al., 2006). Chemokines can induce a conformational switch in the CD11a/CD18 or LFA-1 subunit within a second of contact as demonstrated using an allosteric antibody that reports on the high affinity ligand binding states (Shamri et al., 2005; Green et al., 2006). Neutrophil receptors for chemokine binding such as CXCR1 and CXCR2 are linked to G-protein coupled receptor (GPCR) pathways that activate both CD11b/CD18 or Mac-1 and LFA-1 β_2_-integrins to initiate firm arrest and subsequent migration (Zarbock et al., 2007a). A detailed understanding of how GPCR activation cooperates with signaling via E-selectin ligands on rolling and arresting PMNs is only now emerging (Simon et al., 2000a; Zarbock et al., 2007b). These integrins once activated to a high affinity state can bind ligand and themselves initiate outside-in signals to remodel the cytoskeleton facilitating the next step in the process of pseudopod extension and transendothelial migration (Alon and Ley, 2008).

## Engagement of selectins and GPCRs cooperate in mediating stable adhesion of PMN

Engagement of GPCRs activates Phospholipase C (PLC), which then mobilizes Inositol-1,4,5 triphosphate (IP_3_) and Diacylglycerol (DAG) that triggers an elevation in intracellular calcium level through release of PLC dependent ER stores (Hellberg et al., 1996; Kinashi, 2005). Pharmacological inhibition of PLC in neutrophils, monocytes, and platelets completely abrogates integrin activation downstream of GPCR signaling (Schaff et al., 2008; Graham et al., 2007; Hyduk et al., 2007; Zarbock et al., 2007a). Immediate effector molecules downstream of GPCR and PLC signaling are the Rho GTPases, Rap-1 and cytoskeletal modulators including Talin1, all of which regulate integrin affinity and clustering following ligand binding (Calderwood et al., 1999; Boettner and Van Aelst, 2009). Signaling through GPCRs and DAG activates a Guanine Exchange Factor (GEF), that is denoted CalDAG-GEF1, which in turn activates Rap-1 and modulates Talin1-β_2_ integrin association (Shimonaka et al., 2003; Kinashi et al., 2004; Ghandour et al., 2007; Pasvolsky et al., 2007; Lim et al., 2010). Upon binding of the Talin1 head domain to cytoplasmic sites of the β-integrin tail, a conformational shift is induced that allows the α and β subunits of LFA-1 to move apart and shift to an extended conformation (Calderwood et al., 1999; Kim et al., 2003). A second event significant to converting a rolling PMN to arrest is Rap-1 mediated recruitment of another effector molecule, RapL, to the α cytodomain that facilitates clustering of high affinity LFA-1 (Katagiri et al., 2003).

Activation of integrins can also be achieved by engagement and rolling on selectins, which facilitates the initial capture of leukocytes on the endothelial surface (Ley, 2002; Simon et al., 2000a). Specifically, E-selectin, P-selectin, and L-selectin are critical to leukocyte and lymphocyte capture and rolling through PSGL-1 and other glycosylated ligands. While E and P-selectin are expressed on the endothelium, L-selectin is expressed only on leukocytes and is involved in secondary capture of neutrophils during recruitment (Taylor et al., 1996; Dwir et al., 2001). Selectins form adhesive catch bonds with their glycosylated ligands with high on and off rates and require a threshold level of hydrodynamic shear stress to support rolling and subsequent signaling (Thomas et al., 2002; McDonough et al., 2004; Zhu and McEver, 2005). E-selectin binding to PSGL-1 activates tyrosine kinase Syk and MAPK, which together signal a shift in LFA-1 conformation to an extended and intermediate affinity state (Simon et al., 2000b; Zarbock et al., 2007a). This intermediate affinity state in LFA-1 facilitates deceleration of neutrophil rolling on the endothelium and can trigger firm arrest in the presence of a sufficient density of ICAM-1 (McDonough et al., 2004; Green et al., 2006). Rolling on E-selectin is synergistic with signaling via GPCRs in activation of integrin dependent arrest. The mechanism is not completely elucidated, but may involve calcium acting as a secondary messenger to amplify conversion of additional integrins to a high affinity state and facilitate their formation into focal clusters (Campbell et al., 1998; Alon and Feigelson, 2002; Green et al., 2006; Schaff et al., 2008). Recent studies suggest that E-selectin mediated slow rolling and β_2_ integrin activation in neutrophils is dependent on PLCγ2 and PI3Kγ, which are critical regulators of intracellular calcium release (Mueller et al., 2010). These investigations highlight the cooperativity between chemokines, selectins, and the presence of hydrodynamic shear force for optimum activation of integrins through bi-directional signaling to support a migratory cell phenotype (Simon and Green, 2005).

## Calcium: a temporal and spatial cue for PMN adhesive functions

Calcium (Ca^2+^) is a versatile signaling molecule that is critical to synchronizing rolling, arrest and polarization events during leukocyte migration. Ca^2+^ transients are spatially and temporally regulated by communication between the calcium stores in the endoplasmic reticulum (ER) and membrane distributed calcium channels activated through GPCR signaling and integrin engagement with their ligands on the endothelium. We have mentioned above how chemokine activation through GPCRs is followed by an intracellular Ca^2+^ burst mediated through PLCs that is necessary to trigger integrin activation and leukocyte arrest. This Ca^2+^ flux serves to activate downstream messengers that include calpain, calmodulin, GTPases, and Talin1, some of which also regulate superoxide production, and exocytosis of secretory granules containing additional integrins and proteolytic enzymes (Truneh et al., 1985; Ginis and Tauber, 1990; Smith et al., 1990; Franco et al., 2004; Brechard et al., 2008). Engagement of Mac-1 and LFA-1 can themselves trigger Ca^2+^ transients in the cytosol and activate downstream Ca^2+^ dependent kinases that recruit cytoskeletal proteins necessary for migratory function (Marks and Maxfield, 1990; Jaconi et al., 1991; Hellberg et al., 1995, 1996; Pettit and Hallett, 1997). For example, Ca^2+^ transients are required for neutrophil migration on fibrinogen and fibronectin through Mac-1 engagement and are also important for cell adhesion of platelets, lymphocytes, fibroblasts, and endothelial cells (Su et al., 2000; Schaff et al., 2008).

**Figure 1:**
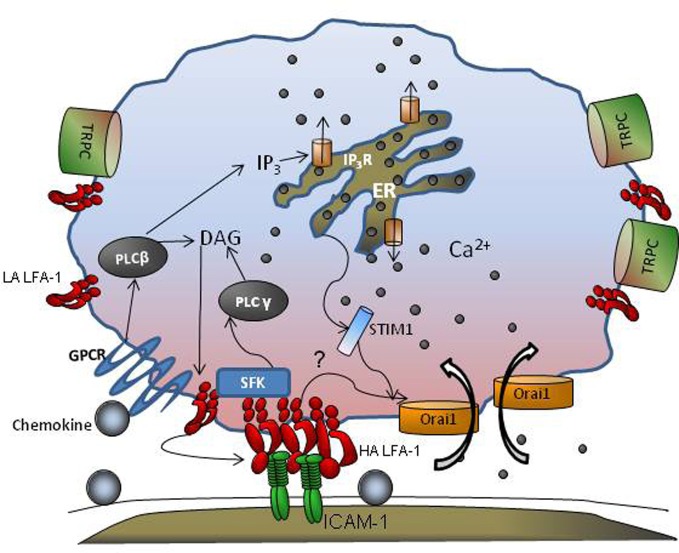
**GPCR and CRAC cooperate during leukocyte adhesion.** Engagement of GPCRs by chemokines activates PLCβ that is cleaved into DAG and IP_3_. While DAG remains membrane bound, IP_3_ is released into the cytosol that then binds to IP_3_ receptors in the endoplasmic reticulum (ER) liberating stores and leading to a rise in cytosolic Ca^2+^ concentration. Integrin receptors shift from low affinity (LA) to high affinity (HA) in response to GPCR and increase their mobility in the membrane. STIM1 senses ER store depletion, binds to the ER membrane, and provides an anchor for transmembrane Orai1 and TRPC channels that cluster and facilitate local Ca^2+^ influx at the plasma membrane. Src family Kinases (SFKs) are recruited to nascent HA LFA-1, which promote clustering, and in turn activate PLCγ which elicits additional ER dependent Ca^2+^ release. Tension on focal clusters of LFA-1/ICAM-1 bonds may also engage cytoskeletal adaptor proteins that activate CRAC mediated calcium influx further promoting integrin clustering and bond formation within a region of adhesive contact we denote the *inflammatory synapse*.

Studies employing multi-channel fluorescence microscopy have provided insight into the spatial and temporal regulation of Ca^2+^ bursts that facilitate cell migration. Using fast confocal laser scanning technology, global cytosolic waves of Ca^2+^ have been reported as “puffs” that are initiated at a submicron scale in response to GPCR engagement (Hillson and Hallett, 2007). Imaging Ca^2+^ dynamics using real-time fluorescence microscopy allows detection of calcium regulation during integrin engagement and its role in leukocyte migration. There are two components of the Ca^2+^ flux signal; a rapid release from ER stores in response to GPCR activation, followed by a slower entry of Ca^2+^ via calcium release activated channels (CRACs) that is mediated by both transient receptor potential (TRP) channels and Orai1, 2, and, 3 that control store operated calcium entry (SOCE; Figure 1). Human neutrophils possess TRPC 1,3 4, and 6, while only TRPC 6 mediates SOCE following E-selectin and GPCR engagement (Heiner et al., 2003; Itagaki et al., 2004; McMeekin et al., 2006). Orai1 CRAC appears to cooperate with these TRPC's to activate calcium influx in human neutrophils (Brechard et al., 2008). The coupling between ER and plasma membrane CRAC to modulate SOCE has recently been shown to involve STIM and Orai proteins (Luik et al., 2006; Brandman et al., 2007; Parvez et al., 2007). STIM1 is a single spanning membrane protein with an unpaired Ca^2+^ binding EF-hand domain that functions as a sensor of ER luminal Ca^2+^, and dynamically redistributes to position the ER proximal to Orai1 spanning the plasma membrane. The association between STIM1 and Orai1 in sensing ER depletion and communicating with the CRAC channel has been elegantly demonstrated using tools that include siRNA mediated knockdown, real time FRET and immunofluorescence imaging (Roos et al., 2005; Brandman et al., 2007; Brechard et al., 2008). STIM1 thus facilitates organized clustering, and conformational changes in TRP and Orai1 to allow Ca^2+^ entry through these channels (Zhang et al., 2005; Wu et al., 2006; Navarro-Borelly et al., 2008). Orai1 is uniformly distributed throughout the plasma membrane in unactivated cells and is the predominant CRAC channel that colocalizes with STIM1 upon ER store depletion (Luik et al., 2006; Wu et al., 2006). Orai1 mediated Ca^2+^ flux was first shown to be critical for T cell function and formation of the immunological synapse, and subsequently its role in Ca^2+^ regulation was identified in B cells, mast cells, and neutrophils (Hoth and Penner, 1992; Feske et al., 2006; Gwack et al., 2008; Schaff et al., 2009). In the context of neutrophil recruitment, we have reported that Orai1 is the predominant CRAC that synchronizes the transition from cell rolling to arrest by cooperating with IP_3_ gated channels downstream of PLC activation (Figure 1). Orai1 CRAC cooperates with other TRP channels on the membrane to mediate Ca^2+^ entry in neutrophils (Brechard et al., 2008). Orai1 mediated Ca^2+^ influx is emerging as a mechanism for signal transduction via mechanical force as tension is transduced intracellularly by high affinity LFA-1 bond clusters during neutrophil arrest. It is reported that tensile force actively stabilizes high affinity LFA-1 bonds during the transition from rolling to arrest (Green et al., 2006; Alon and Dustin, 2007) A putative mechanism is one in which Orai1 and high affinity LFA-1 become colocalized during bond formation with ICAM-1 (Dixit et al., 2011). In this manner, integrin mediated local Ca^2+^ flux enhances integrin contact with the endothelium by promoting cytoskeletal redistribution that engage and anchor integrin cytodomains (Cinamon et al., 2001; Dixit et al., 2011). Local Ca^2+^ at these sites reinforces adhesion by recruiting additional clusters of LFA-1. This process may explain why high affinity LFA-1 clusters bound to ICAM-1 are observed both at the uropod and at the base of newly forming pseudopods as PMN adopt a polarized morphology and migrate perpendicular to the direction of blood flow (Dixit et al., 2011). F-actin is also found enriched at these sites of high PMN traction (Smith et al., 2007; Schaff et al., 2009; Dixit et al., 2011). In the absence of the shift from intermediate to high affinity LFA-1, there is insufficient localization and recruitment of Orai1 to LFA-1 sites leading to decreased intracellular Ca^2+^ flux (Dixit et al., 2011). In the absence of stable high affinity LFA-1/ICAM-1 bonds, impaired F-actin polymerization is also observed and the processes of neutrophil polarization and transmigration are abolished. The role of local Ca^2+^ flux in the timing of cell arrest-polarization-migration has been elucidated by real-time imaging of Ca^2+^ flux. Using flash lamp-based excitation, it was shown that Ca^2+^ transients cycling at 6 μ s intervals were associated with Myosin-II activation during uropod retraction (Clark and Petty, 2008). In addition, we have shown that engagement of high affinity LFA-1 clusters and shear stress are critical to initiation of Ca^2+^ influx during arrest (Dixit et al., 2011). Taken together, these data suggest that neutrophils rolling to arrest utilize focal adhesions as mechano-sensors that convert shear stress mediated tensile force into local bursts of Ca^2+^ influx that promotes cytoskeletal engagement, and an adhesion strengthened and migratory phenotype.

## Cytoskeletal organization at integrin cytodomains

An important question that has emerged is what are the cytodomain linkages that transduce force intracellularly from the high affinity bonds between β_2_-integrin and ICAM-1? Furthermore, how does neutrophil polarity and directional migration become responsive to the magnitude and direction of shear stress? The earliest steps in neutrophil recruitment are chemokine activation of GPCRs that triggers integrin activation and initiates linkage to the cytoskeleton at relatively low levels of cytosolic Ca^2+^ (i.e., ~100 nM; Lum et al., 2002; Green et al., 2006). Cytoplasmic adaptor proteins including Kindlin-3 and Talin1 build up at the integrin cytodomain, as high affinity clusters of integrins accumulate on a rolling neutrophil, even before integrin bonds form focal adhesions at the inflammatory substrate (Lefort et al., 2012). In the case of LFA-1, we have observed that a high affinity state and engagement to ICAM-1 homodimers results in bonds that last ~10-fold longer and transmit 100-fold higher force as compared to monomeric LFA-1/ICAM-1 bonds (Sarantos et al., 2005; Evans et al., 2010). The valence and conformation of the integrin bond in this case can influence the bond lifetime and amount of force that is transduced across the membrane. In this manner, LFA-1 clusters can form adhesion-strengthened complexes that are resistant to dissociation as they link to the nascent cytoskeleton leading up to migration (Astrof et al., 2006; Alon and Dustin, 2007; Puklin-Faucher and Sheetz, 2009). The β tail of integrins acts as a scaffold for binding cytoskeletal adaptor proteins, as well as tyrosine kinases such as Src Family Kinases (SFKs) including Src and Syk that signal to activate and cluster more integrins at the contact site (Obergfell et al., 2002). SFKs associate rapidly with the LFA-1 cytodomain and can regulate integrin affinity, avidity, and subsequent signaling to the cytoskeleton to initiate cell spreading (Roskoski, 2004; Arias-Salgado et al., 2005; Sarantos et al., 2008). Genetic deletion and inhibition of SFKs in neutrophils abrogates rearrangement of high affinity LFA-1 clusters along the uropod-pseudopod axis and impairs co-clustering of high affinity CD18 with F-actin during polarization (Piccardoni et al., 2004; Sarantos et al., 2008). Not only is slow rolling on E-selectin abolished in Syk deficient bone marrow chimeric mice, but these mice also exhibited impaired integrin mediated signaling, defective respiratory burst, degranulation, and spreading in response to inflammatory stimuli (Mocsai et al., 2002; Zarbock et al., 2007a). Thus, Src and Syk tyrosine kinases appear to function in events both leading up to LFA-1 ligand engagement and signaling of subsequent effector functions. More research on their discrete functions during PMN migratory responses is needed.

**Figure 2:**
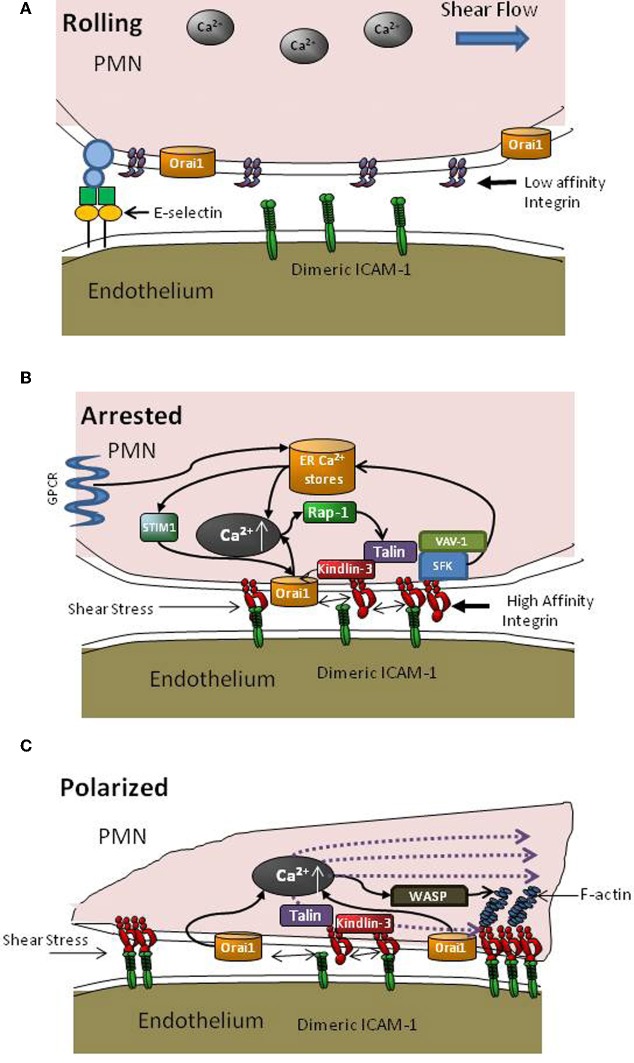
**Intracellular signaling events supporting PMN recruitment. (A)** During PMN capture and rolling on inflamed endothelium, β_2_-integrins are randomly distributed on the plasma membrane predominantly at low affinity and a low basal level of intracellular Ca^2+^ is maintained. **(B)** Transition from rolling to arrest involves activation via GPCR signaling that elicits Ca^2+^ release via DAG (see Figure 1) and an upshift in LFA-1 to a high affinity state, which promotes bond formation with ICAM-1 on inflamed endothelium. Depletion of ER stores leads to communication with Orai1 CRAC at the membrane via STIM1 proteins. As LFA-1/ICAM-1 bonds take up tensile forces they recruit Kindlin-3 and colocalize with Orai1 to facilitate cooperation with PLC mediated Ca^2+^ flux, which in turn catalyzes recruitment of Rap-1 GTPases and cytoskeletal elements such as Talin to LFA-1 cytodomains to initiate F-actin recruitment and pseudopod projection. **(C)** New pseudopod projection and cell polarization is oriented by the dynamic redistribution of LFA-1/ICAM-1 into macro-clusters, Orai1 mediated Ca^2+^ influx, and assembly of the F-actin cytoskeleton that guides migration in a manner dependent upon direction of shear stress and cytoskeletal force distribution.

There is much recent interest in the roles of Talin1 and Kindlin-3 as key cytoskeletal adaptor proteins in the regulation of integrin affinity and clustering during the transition from neutrophil rolling to arrest and shape polarization as it navigates to sites of transmigration on inflamed endothelium (Sarantos et al., 2008; Puklin-Faucher and Sheetz, 2009; Lefort et al., 2012). How these cytoskeletal proteins bind to the LFA-1 cytodomain as it engages ICAM-1 and transduce signals to guide neutrophil migration under shear flow is beginning to come to light. Talin1 associates with the β2 tail of LFA-1, unclasping the α and β chains to allow a conformational upshift to its ligand-binding state as reported in neutrophils and T cells (Calderwood et al., 1999; Simonson et al., 2006). Talin clusters with LFA-1 at the immunological synapse and also localizes at focal adhesions with LFA-1 in leukocytes along with paxillin, which provides its main linkage to F-actin during assembly in response to a local cytosolic gradient of Ca^2+^ (Lum et al., 2002). Kindlin-3 also binds to integrin β-tails and has been shown to play a role in GPCR activated upshift in integrin affinity and subsequent leukocyte adhesion on the endothelium (Moser et al., 2009; Svensson et al., 2009). This protein was recently identified as the key molecule defective in leukocyte adhesion deficiency III (Mory et al., 2008). Talin and Kindlin-3 recognize two distinct binding sites on the LFA-1 cytoplasmic tail and cooperative binding may be requisite for assembly of a high affinity LFA-1 that is competent to form multivalent bond clusters with ICAM-1 (see Figure 2) (Moser et al., 2009). Talin and Kindlin-3 are critical for adhesion strengthening and cell spreading under shear stress at a step downstream than affinity regulation as shown in studies of β_1_ integrin (Feigelson et al., 2011; Hyduk et al., 2011). Kindlin-3 has been carefully studied in T-cell receptor mediated outside-in stabilization of chemokine activated LFA-1 bond formation with ICAM-1. It was shown to associate with RACK1 at the cytodomain in order to effect clustering of LFA-1 (Feigelson et al., 2011; Feng et al., 2012). The observation that α_*II*_β_3_ receptors on Kindlin-3 deficient platelets activated to high affinity by Mn^2+^ can bind to fibrinogen coated substrates, but downstream cell spreading is severely impaired, indicates that integrin mediated cytoskeletal rearrangement through outside-in signaling is defective (Moser et al., 2008). Thus, Kindlin-3 is critical for stabilization and downstream transduction events necessary for adhesion strengthening through β_3_ subunits. In the case of β_2_-integrin, Kindlin-3 association precedes recruitment of Talin to the β-subunit of LFA-1 in a pathway that involves GPCR activation, Ca^2+^ flux, Rap-1 recruitment, and Phosphatidylinositol 4,5 bisphosphate (PIP_2_) activation (Di Paolo et al., 2002; Puklin-Faucher and Sheetz, 2009; Lefort et al., 2012). However, the significance of Ca^2+^ influx through CRAC and cooperation with GPCR release of ER stores for initial Kindlin-3 association is yet to be elucidated. Our laboratory has been examining the role of Kindlin-3 in PMN arrest and adhesion strengthening in shear flow. We observe that Kindin-3 binding to the β-subunit of LFA-1 is critical for adhesion strengthening of arrested PMN at high shear stress and facilitates rapid clustering of LFA-1 at focal sites that engage ICAM-1. This data thus supports previous studies highlighting Kindlin-3 as a key player in mediating adhesion strengthening through β_1_ integrins and its dispensable role in GPCR mediated integrin affinity upshift (Hyduk et al., 2011). Transmission of tensile force provided by blood flow to the cytodomain of LFA-1 activates a local burst of Ca^2+^ via Orai1 CRAC that cooperates with ER stores to effect a local burst in Ca^2+^ concentration (Figure 2). Kindlin-3 appears to be critical at this step, since knockdown of Kindlin-3 expression using lentiviral transfection of shRNA abrogates its physical association with high affinity LFA-1 and Orai1. Thus, Kindlin-3 may serve a critical role as an adaptor molecule whose binding to the LFA-1 cytodomain requires a force sensitive allosteric step that allows binding directly or in a complex with as yet unidentified proteins to open proximal Orai1 channels. This linkage between high affinity LFA-1/ICAM-1 bonds and Orai1 via Kindlin-3 begins to explain how it serves as an adaptor in mediating focal clustering of LFA-1 that supports adhesion strengthening (Dixit et al., 2011 Figure 2). Precisely how Kindlin-3 communicates with Orai1 and what the role of other cytoskeletal proteins such as Talin, paxillin and vinculin associated with focal LFA-1 clusters in this process are under study in our laboratory.

## Leukocyte signaling in disease

Remarkable is the high frequency of immunodeficiency diseases that are associated with mutations in the effector molecules that directly influence affinity modulation and clustering of integrins. These include Orai1, Kindlin-3, WASp, CalDAG-GEF1, and Vav1, all of which have been identified in leukocyte adhesion deficiencies. Moreover, all of these components cooperate with Ca^2+^ mediated signaling of adhesion stabilization and integrin outside-in signaling (Marks and Maxfield, 1990; Sjaastad and Nelson, 1996). CRAC channels and their crosstalk with ER stores of Ca^2+^ are critical to facilitating F-actin polymerization and integrin polarity during migration (Schaff et al., 2009; Dixit et al., 2011). Recently, a point mutation in the Orai1 gene at the R91W locus was discovered to be associated with a severe immunodeficiency in patients. This mutation is clinically manifested by infections in childhood, ectodermal dysplasia, and congenital myopathy (Feske et al., 2006). These symptoms were similar to those observed in SCID (severe combined immunodeficiency disease) patients except that total lymphocyte counts were normal in Orai1 deficient patients as compared to SCID (Feske, 2009). This missense mutation in Orai1 did not interfere with interactions between Orai1 and STIM1, which suggest that the immunodeficiency is derived from defective Orai1 driven Ca^2+^ flux. Blocking SOCE with CRAC channel inhibitors, using siRNA, or genetic deletion to knockdown Orai1 expression all result in impaired neutrophil arrest, polarization, and abrogation of directional migration under shear flow (Schaff et al., 2009; Dixit et al., 2011). Furthermore, Ca^2+^ entry through Orai1 and STIM1 drives focal adhesion turnover through Ras and Rac1 and together play a vital role in tumor metastasis (Yang et al., 2009). This critical role of calcium in regulating cellular adhesive processes makes it an attractive therapeutic target to reduce pro-inflammatory responses in specific leukocyte subsets.

Immunodeficiencies have also been linked to impaired GTPases, GEFs, and cytoskeletal protein signaling. Leukocyte adhesion deficiency I, II, and III occur due to defects in β_2_ integrin structure, mutations in the fucosyl transporter gene required for producing sialyl-Lewis^*x*^ selectin ligands that support leukocyte rolling on the endothelium, and a general defect in integrin activation of β_1_, β_2_, and β_3_ integrins, respectively (Abram and Lowell, 2009) Cytoskeletal proteins such as Talin1 and Kindlin-3 provide activation and stabilization signals when bound to cytoplasmic domains of integrins (Zhang et al., 2008; Hyduk et al., 2011). Upstream of these proteins, integrin activation is controlled by GTPases such as Rap-1 and its GEF, CalDAG-GEF1 which function downstream of GPCR activation (Pasvolsky et al., 2007; Mory et al., 2008). Mutations in Kindlin-3 are responsible for LAD III related integrin activation defects contributing to recurrent bacterial infections, impaired healing of wounds, defects in platelet activation and severe bleeding tendencies (Abram and Lowell, 2009). Mutations in CalDAG-GEF1 were also found present in a subset of LADIII patients and re-expression of CalDAG-GEF1 was unable to rescue the LADIII phenotypic defects (Svensson et al., 2009). In comparison, re-expression of the Kindlin-3 protein in immortalized lymphoblast cell lines derived from patients restored their adhesive and migratory defects (Abram and Lowell, 2009; Malinin et al., 2009; Svensson et al., 2009). This implicates Kindlin-3 as the key defective protein underlying LADIII manifestation.

Similar to Kindlin-3, WASp also connects the actin cytoskeleton to integrin cytodomains to facilitate leukocyte migration via control of integrin adhesion functions. A crucial effector of Rho GTPases and an important activator of the Arp2/3 cytoskeletal complex, WASp deficiency leads to Wiskott-Aldrich syndrome that is characterized by increased susceptibility to infections (Thrasher, 2002). We reported that a defect in WASp in both mice and human is associated with impaired clustering of β_2_-integrins and severely impaired adhesion and migration of neutrophils on inflamed endothelium (Zhang et al., 2006). WASp deficiency contributes to defective T cell trafficking toward a chemokine gradient, revealing its profound role in signaling through GPCR pathways and guiding leukocyte migration (Snapper et al., 2005). Many other signaling proteins associating with integrins such as Rho family of GTPases, P21 activated kinases (PAKs) and their effector molecules are now emerging as significant contributors to inflammatory disorders and cancer progression (Ahn et al., 2011; Yoon et al., 2011). These molecules are all activated downstream of GPCR engagement and assist in strengthening integrin bond clusters required for leukocyte pseudopod extension and eventual recruitment to sites of insult.

## Conclusion and perspectives

With each heartbeat, leukocytes make a fateful decision when they encounter vascular sites of inflamed endothelium; to arrest or not to arrest. This singular event multiplied by millions of encounters can determine the intensity of the neutrophilic response to infectious or autoimmune tissue insults. Assisting in this decision process is the relative density of chemokines and selectins expressed on inflamed endothelium that facilitate neutrophil activation by ligating their respective cognate receptors on the tethered cell. In this review, we detailed how cytosolic release of Ca^2+^ converges with influx through CRAC, thereby providing a means to dynamically modulate the number and location of integrin bonds and subsequent migration. This is accomplished by shear stress mediated tensile force transmission, which requires bond formation at sites in which β_2_-integrins are engaged at sufficient bond strength and density where their survival is ensured. At these locations, high affinity LFA-1 associates with cytosolic Kindlin-3 thus enabling association of a complex with Orai1 that together transduce a local increase in Ca^2+^. This in turn activates membrane diffusion of additional high affinity LFA-1 to bond with available endothelial ICAM-1. Further, local cytosolic release of Ca^2+^ promotes the assembly of cytoskeletal elements including Talin and F-actin to the integrin tail in a complex that provides the machinery for adopting a polarized elongated shape as a neutrophil extends pseudopods and initiate transendothelial migration. In this manner, mechano-transduction through integrins provides a means for sensing the direction and magnitude of shear force via a complex that involves at a minimum LFA-1, Orai1, Kindlin-3, Talin1, Vav-1, and WASp. These molecules enable neutrophils to efficiently navigate the journey from the blood stream to inflammatory sites that is critical for host defense.

### Conflict of interest statement

The authors declare that the research was conducted in the absence of any commercial or financial relationships that could be construed as a potential conflict of interest.
